# Pharmacological STING Activation Is a Potential Alternative to Overcome Drug-Resistance in Melanoma

**DOI:** 10.3389/fonc.2020.00758

**Published:** 2020-05-14

**Authors:** Sandhya Chipurupalli, Raja Ganesan, S. P. Dhanabal, M. Suresh Kumar, Nirmal Robinson

**Affiliations:** ^1^Cellular-Stress and Immune Response Laboratory, Center for Cancer Biology, University of South Australia, Adelaide, SA, Australia; ^2^Department of Pharmacology, JSS College of Pharmacy, JSS Academy of Higher Education & Research, Ooty, India; ^3^TIFAC CORE in Herbal Drugs, JSS College of Pharmacy, JSS Academy of Higher Education & Research, Ooty, India

**Keywords:** STING, NRF2, diABZI, BRAF, melanoma, dabrafenib, vemurafenib

## Abstract

Melanoma is the most aggressive type of skin cancer and resistance to the conventional chemotherapy is the major cause for its poor prognosis. Metabolic perturbations leading to increased production of reactive oxygen species activate NRF2-dependent anti-oxidative responses to survive oxidative stress. This protective function of NRF2 is the primary cause for therapy resistance in cancer as anti-cancer agents such as BRAF inhibitors also induce NRF2-dependent antioxidative response. We had reported that type I interferons produced upon activation of STING, abrogates NRF2 function. Therefore, we investigated if STING agonists such as the newly developed dimeric aminobenzimidazole (diABZI) could sensitize melanoma cells to the clinically used BRAF inhibitors. Our results reveal that pharmacological activation of STING by diABZI, down regulates NRF2-dependent anti-oxidative responses and potentiates cell-death in melanoma cells when used in combination with BRAF inhibitors.

## Introduction

Melanoma is the most aggressive type of skin cancer and is responsible for most skin cancer related deaths (1). About 50% of melanomas have activating *BRAF* mutations (*BRAF*V600E) leading to enhanced proliferation and survival (2). *BRAF* mutation is involved in various mechanisms of melanoma progression but predominantly hyperactivates downstream MEK/ERK pathway (3). Surgical excision, targeted therapy, immunotherapy and chemotherapy are the current therapeutic options for the melanoma patients (4). Targeted therapies include BRAF and MEK inhibitors. Vemurafenib, was the first FDA approved specific BRAF inhibitor (BRAFi) (3). Two years later, Dabrafenib, another BRAFi was approved by FDA which has higher potency and fewer side effects than Vemurafenib (5). These two specific BRAFis show excellent clinical response with substantial reduction of tumor burden in the initial stages. However, the long-term success is compromised due to the development of drug resistance (6).

Re-activation of the MAPK pathway is the major cause for the development of drug resistance to the BRAFi. Although BRAFis are efficient in decreasing cell proliferation via inhibition of MAPK/ERK activation, reactivation of this pathway occurs in 80% of the BRAFi-resistant cancer cells suggesting that these cells rapidly adapt to MAPK inhibition (7). In addition, melanoma cells undergo metabolic adaptations to cope with reactive oxygen species (ROS)-induced damage. NRF2 (Nuclear factor (erythroid-derived 2) -like 2) is a transcription factor which regulates anti-oxidative response in response to ROS and protects against oxidative damage. In melanoma NRF2 augments hexose monophosphate shunt (8, 9) and this metabolic adaptation contributes to the intracellular redox balance and allows the BRAFi-resistant melanoma cells to survive under oxidative stress (9).

We had recently shown that type I IFNs (IFN-I) negatively regulate Nrf2 response through receptor-interacting protein kinase (RIPK) signaling during infection (10). The induction of IFN-I in response to infection is primarily mediated by Cyclic GMP-AMP synthase (cGAS)-Stimulator of interferon genes (STING) pathway. Interestingly, cGAS-STING activation has been considered as a therapeutic strategy for cancer (11, 12). STING is a transmembrane protein present on endoplasmic reticulum (ER) and is activated when the cGAS (cyclic-GMP-AMP-synthase) senses cytosolic double stranded DNA and converts it into cyclic dinucleotides (CDNs) which directly binds to STING. STING then translocates from endoplasmic reticulum to the perinuclear region (13) where, it oligomerizes with TANK-binding kinase-1 (TBK1) resulting in the phosphorylation of STING and the transcription factor IRF3 to induce IFN-I and other cytokines (14, 15). Thus, the enhanced expression of IFN-I mediates the cytotoxic effects (16). However, recent studies have shown that there is a recurrent loss of STING-activity in melanoma cells and are incapable of producing IFN-I when exposed to cytosolic DNA (17).

We hypothesized that activation of NRF2 in BRAFi-resistant melanoma cells could be the cause of diminished STING-activity. Hence, we investigated the ability of a recently discovered small molecule STING agonist, dimeric amidobenzimidazole (diABZI) (18) to circumvent the BRAFi-resistance developed by melanoma cells. We show that pharmacological activation of STING using diABZI downregulates NRF2-dependent antioxidative responses thereby sensitizing melanoma cells to BRAFis.

## Materials and Methods

### Cell Culture

C32 and SK-MEL-28 cells were obtained from the laboratory of Claudine Bonder, Centre for Cancer Biology, University of South Australia and were cultured in RPMI medium supplemented with 10% fetal bovine serum and maintained at 37°C, 5% CO_2_.

### Drugs and Treatments

BRAF inhibitors Dabrafenib (Cat No. HY-14660), Vemurafenib (Cat No. HY-12057) and diABZI STING agonist-1 trihydrochloride (Cat No. HY-112921B) were procured form MedChem Express. CDDO-methyl ester (SMB00376) was purchased from Sigma Aldrich and used at a concentration of 500 nM. Dabrafenib, Vemurafenib and diABZI were used at their given IC_50_ concentrations 0.6, 31, and 21 nM respectively.

### Immunoblotting

C32 or SK-MEL-28 cells were lysed in radioimmunoprecipitation assay (RIPA) buffer supplemented with protease and phosphatase inhibitors. Protein concentrations were estimated using Pierce BCA Protein assay kit (Thermo Fisher Scientific), as per the instructions. Equal amounts of proteins were separated on 4–20% Mini-PROTEAN TGX Stain-Free Gels (#4568094, Bio-rad). Proteins were then transferred onto PVDF membranes and probed with the following antibodies: STING/TMEM173 (NBP2-24683, Novus), phospho-STING (#19781, Cell Signaling technology), TBK1 (#3504, Cell Signaling Technology), phospho-TBK1 (#5483, Cell Signaling Technology), NRF2 (ab137550, Abcam). Beta actin or Calnexin were used as loading controls. After incubation with secondary horseradish peroxidase (HRP)-conjugated antibodies, the blots were washed and developed using enhanced chemiluminescence reagent and imaged in the ImageQuant LAS4000.

### Immunofluorescence Staining and Confocal Microscopy

**Figure 1:**
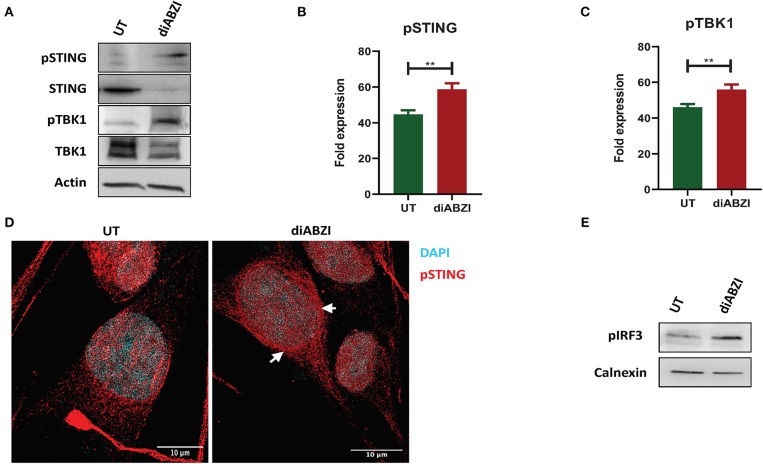
diABZI activates STING in melanoma cells. **(A)** Immunoblot analysis of STING pathway in untreated vs. diABZI treated samples (*n* = 3). **(B)** Densitometric analysis of phosphorylated STING (pSTING) and **(C)** Densitometric analysis of phosphorylated TBK1 (pTBK1) (*n* = 3). **(D)** Immunofluorescence staining and confocal microscopy analysis of pSTING in untreated vs. diABZI treatment. Arrows indicate perinuclear localization of pSTING. **(E)** Immunoblot of phosphorylated IRF3 in diABZI treated samples. Scale bar = 10 μm; **P < 0.01.

**Figure 2:**
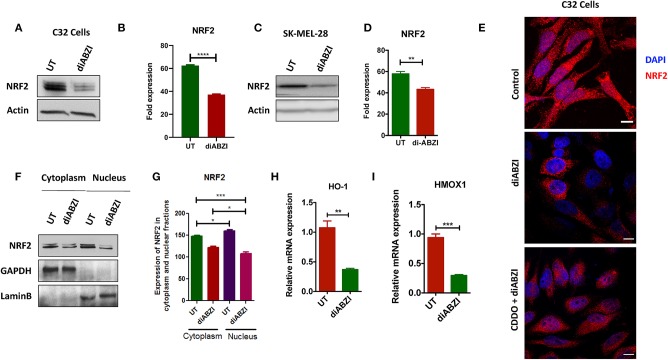
diABZI prevents NRF-2 activation in melanoma cells. **(A)** Immunoblot analysis of NRF2 upon diABZI treatment in C32 cells and **(B)** densitometric quantification of NRF2 in untreated vs. diABZI treatment (*n* = 3). **(C)** Immunoblot analysis of NRF2 upon diABZI treatment in SK-MEL-28 cells and **(D)** densitometric quantification of NRF2 in untreated vs. diABZI treatment (*n* = 3). **(E)** Immunofluorescence staining and microscopic analysis of NRF2 accumulation in the nucleus. **(F)** Immunoblot of cytosolic and nuclear cell fractions of untreated and diABZI treated C32 cells. **(G–I)** Relative mRNA expression of NRF2 downstream target genes. Relative mRNA expression levels of *HO-1* **(F)** and *HMOX1* **(G)**. Scale bar = 10 μm; **P < 0.01, ***P < 0.001, ****P < 0.0001.

**Figure 3:**
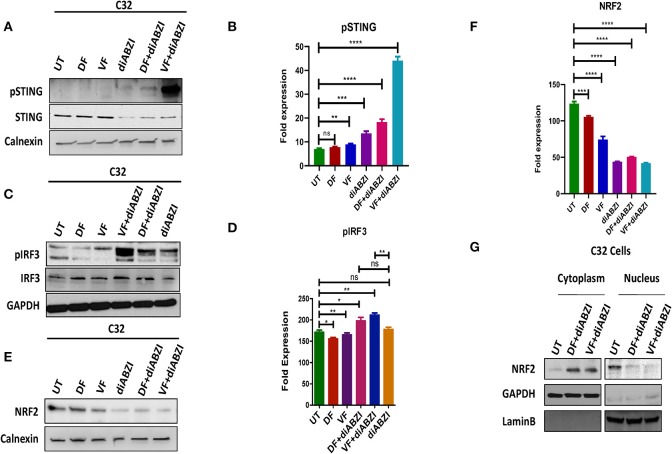
Combination of diABZI and BRAF inhibitors prevent NRF2 activation and activate STING. **(A)** Immunoblot analysis of phospho STING upon treatment with diABZI and BRAFis, Dabrafenib (DF) and Vemurafenib (VF). **(B)** Densitometric quantification of pSTING (*n* = 3). **(C)** Immunoblot analysis of phospho IRF3 upon treatment with diABZI and BRAFis, Dabrafenib (DF) and Vemurafenib (VF). **(D)** Densitometric quantification of pIRF3 (*n* = 3). **(E)** Immunoblot analysis of NRF2 upon diABZI and BRAFi treatment in C32 cells. **(F)** Densitometric quantification of NRF2 (*n* = 3). **(G)** Immunoblot of cytosolic and nuclear cell fractions upon treatment with diABZI and BRAFis, Dabrafenib (DF) and Vemurafenib (VF). **P < 0.01, ***P < 0.001, ****P < 0.0001.

C32 cells grown on the glass coverslips were treated with BRAFis and diABZI for 24 h and fixed with 4% formaldehyde in PBS for 15 min at RT. The cells were then permeabilized with 0.3% Triton X-100 in PBS for 5 min and then blocked with 3% BSA for 1 h at RT. The cells were incubated overnight with primary antibody against NRF2 and pSTING at 4°C. After overnight incubation, the cells were washed with PBS and incubated with Alexa Fluor 594-conjugated goat anti-rabbit secondary antibody for 1 h at RT in the dark. The cells were then washed, and coverslips were mounted using ProLong Diamond antifade containing DAPI to stain the nuclei. The cells were imaged under Leica SP8 confocal or Leica THUNDER imager. Images taken in (Figures 2C, 3E) using Leica THUNDER imager were processed using computational clearing tool. The image in (Figure 1D) was acquired using Leica SP8 and processed by on-the-go deconvolution LIGHTNING tool.

### Quantitative Real-Time PCR

Total RNA from C32 cells (1 × 10^6^ cells/well) was isolated using RNeasy Mini kit (74106; Qiagen), and 500 ng cDNA was synthesized with random hexamers by reverse transcription (SuperScript III; 18080; Invitrogen). Twenty microliters of PCR reactions contained 10 ng cDNA, 0.4 μmol/liter of each forward and reverse primer, and master mix (SsoFast EvaGreen Supermix; 1725201; Bio-Rad). Real-time PCR was performed under the following conditions: initial denaturation step at 95°C for 2 min and 40 cycles at 95°C for 5 s and 60°C for 15 s, followed by a denaturation step at 95°C for 60 s and a subsequent melt curve analysis to check amplification specificity. Results were analyzed by the comparative threshold cycle method with hypoxanthine-guanine phosphoribosyltransferase (*HPRT*) as the endogenous reference gene for all reactions. The relative mRNA levels of untreated samples were used as normalized controls for the diABZI treated samples. All reactions were performed in triplicate and a non-template control was included in all experiments to exclude DNA contamination. Primer sequences are listed in (Table 1).

**Table 1:** Primers used in the study.

**Primers**	**Sequence (5' to 3')**
HO-1 (forward)	ATGACACCAAGGACCAGAGC
HO-1 (reverse)	GTGTAAGGACCCATCGGAGA
HMOX1 (forward)	CTCAAACCTCCAAAAGCC
HMOX (reverse)	TCAAAAACCACCCCAACCC
HPRT (forward)	CATTATGCTGAGGATTTGGAAAGG
HPRT (reverse)	CTTGAGCACACAGAGGGCTACA

### Time-Lapse Imaging

C32 cells were seeded (2 × 10^4^ cells/well) in the ibidi 8-well chamber slide (cat no. 80841) suitable for live cell imaging in the CellVoyager CV1000 Confocal imaging system (Olympus Life Sciences). A day after, the cells were treated with appropriate concentrations of BRAFis and diABZI and set up for time-lapse imaging over 24 h duration at an interval of 20 min. Images were acquired under 10X magnification.

### Cell Migration Assay

C32 cells were seeded in a 24-well plate and left overnight for the cell monolayer to reach 70–80% confluency. Then without changing the medium, a scratch was created across the center of the cell monolayer in each well using a sterile 10 μL pipette tip. Wells were then washed twice gently with medium to remove the detached cells. Next the wells were replenished with medium containing BRAFis and diABZI. Cell migration and growth towards the center were observed and imaged using NIKON Eclipse Ts2 microscope before and after 24 h treatment to record the cell migration.

### MTS Cell Viability Assay

C32 or SK-MEL-28 cells were cultured in 96-well plate at a density of 10x10^3^. After 16-24 h, cells were treated with BRAFis and diABZI for 24 h. Twenty microliters of MTS reagent was added into each well and incubated for 4 h at 37°C in standard culture conditions. After 4 h of incubation, absorbance of the untreated and treated cells was measured at 490 nm using a microplate reader (Bio Tek^TM^ EPOCH).

### Statistical Analysis

Statistical analyses were performed using GraphPad Prism Software (Version 8.0). Unpaired Student's *t*-test was conducted for all the datasets unless specified otherwise to determine statistical significance. All the data are represented as mean ± SEM. For all tests, a *P-*value < 0.05 was considered statistically significant (^*^p < 0.05; ^**^p < 0.01; ^***^p < 0.001; ^****^p < 0.0001).

## Results

### diABZI Activates STING in Melanoma Cells

Recent studies have reported recurrent loss of STING activity or responsiveness in melanoma cells (17). Therefore, we investigated the effect of diABZI, the novel small molecule STING agonist in C32 melanoma cells harboring BRAF600^VE^ mutation and are moderately resistant to BRAFi (19). Cell lysates collected after 24 h treatment with diABZI (21 nM) were immunoblotted for phospho-TBK1, phospho-STING, TBK1, and STING (Figure 1A) and the band intensities of treated samples relative to untreated were quantified and plotted (Figures 1B,C). Treatment with diABZI resulted in enhanced phosphorylation of TBK1 and STING and increased localization of phospho STING in the perinuclear region (Figure 1D). It also resulted in the phosphorylation of IRF3 (Figure 1E) which suggests activation of STING pathway. However, we also observed downregulation of total STING, which has been shown to be due to its degradation in autophagosomes (20). Consistently, diABZI treatment resulted in increased expression of LC3B suggesting increased autophagy (Figure S1). Collectively, these data show that STING pathway can be activated in melanoma cells using diABZI.

### diABZI Prevents NRF2 Activation in Melanoma Cells

BRAFi-resistant melanoma cells are characterized by enhanced mitochondrial OXPHOS (oxidative phosphorylation) resulting in enhanced oxidative stress. To survive under such stress, BRAFi-resistant melanoma cells activate NRF2 dependent antioxidative responses (9). Therefore, we questioned if the small molecule STING agonist diABZI affects the NRF2-dependent antioxidant response. Immunoblot analysis showed decreased levels of NRF2 upon diABZI treatment in C32 (Figures 2A,B) and SK-MEL-28 cells (Figures 2C,D) which are also known to acquire resistance (21). Microscopical examinations also showed dispersed staining of NRF2 in the cytosol and reduced nuclear accumulation upon diABZI treatment (Figure 2E). However, activation of NRF2 using 2-Cyano-3,12-dioxooleana-1,9-dien-28-oic-acid (CDDO)-methyl ester (Me) (22), resulted in increased nuclear translocation (Figure 2E). Cell fractionation followed by immunoblotting confirmed that the translocation of NRF2 into nucleus was reduced upon STING activation (Figures 2F,G). Moreover, quantitative real time PCR (RT-PCR) revealed that the mRNA expression NRF2-target genes *HO-1* (Figure 2H) and *HMOX-1* (Figure 2I) were significantly downregulated upon treatment with diABZI. These data suggest that NRF2 activity is abrogated upon STING activation.

### Combination of diABZI and BRAF Inhibitors Activate STING and Prevent NRF2 Translocation

Since we observed that diABZI potently activates STING pathway and prevents NRF2 activation, we investigated if the combination of diABZi with the currently available BRAFis; Dabrafenib and Vemurafenib will also activate STING and inhibit NRF2. As speculated, combined treatment of Dabrafenib or Vemurafenib with diABZI, enhanced phosphorylation of STING (Figures 3A,B) and IRF3 (Figures 3C,D). STING activation also lead to reduced levels of NRF2 in C32 (Figures 3E,F) and SK-MEL-28 cells (Figure S2a). Furthermore, cell fractionation and microscopy revealed that combined treatment of diABZI with both BRAFis resulted in reduced nuclear translocation of NRF2. However, reduced nuclear localization was more pronounced when diABZI was combined with Vemurafenib (Figure 3F, Figure S2b).

### diABZI Prevents Cell Proliferation and Migration

**Figure 4:**
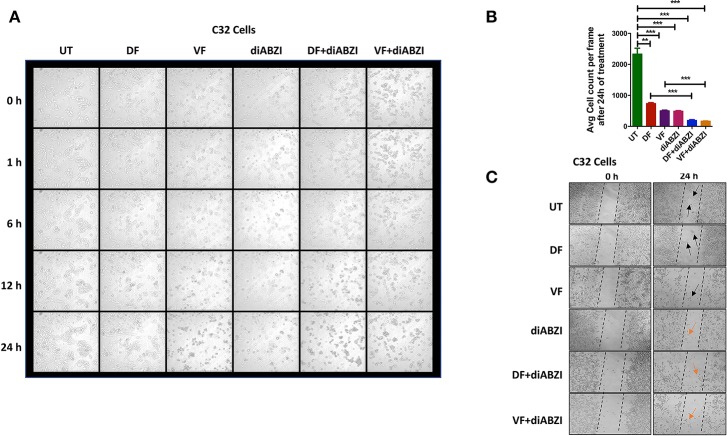
diABZI prevents cell proliferation and migration. **(A)** Time-lapse microscopy showing images acquired at 0, 1, 2, 6, 12, and 24 h of diABZI treatment vs. untreated. **(B)** Quantification of time-lapse images acquired at 24 h of treatment vs. untreated using ImageJ software. **(C)** Scratch assay showing the migration of cells in the untreated compared to the cell migration upon treatment with Dabrafenib (DF), Vemurafenib (VF), diABZI and combination of diABZI with DF and VF. Arrow in black shows the cell migration and closure of the scratch whereas; arrow in orange shows no migration but dead cells.

We next investigated whether the combination of diABZI with BRAFis prevents cell proliferation and migration. Time-lapse imaging of cells treated with diABZI for a duration of 24 h showed remarkable decrease in cell proliferation which was further significantly reduced when combined with BRAFis (Figures 4A,B, Figure S3a and proliferation movies provided as Supplementary Data).

Additionally, we performed *in-vitro* scratch assay to study cell migration which is crucial for cell proliferation and cancer progression. Hence, we created a scratch on confluent cell monolayer and treated the cells with diABZI or diABZI in combination with BRAFis. Dabrafenib and Vemurafenib treatments for 24 h markedly reduced cell migration compared to the untreated cells (scratch was completely closed) (Figure 4C). However, cell death was observed in the diABZI treated cells which affected cell migration and this effect was further enhanced in the combination of diABZI and BRAFis (Figure 4C). Together, these results suggest that diABZI in combination with Dabrafenib or Vemurafenib synergistically prevents melanoma cell proliferation and migration.

### diABZI and BRAF Inhibitors Induced Cell Death Is NRF2 Dependent

**Figure 5:**
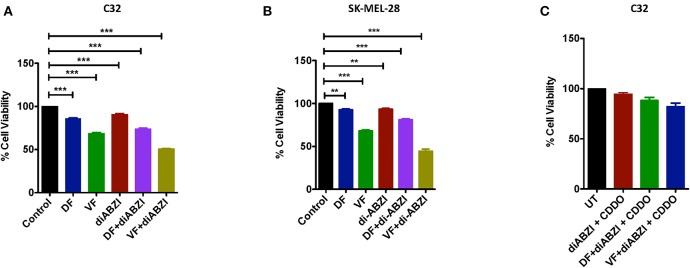
diABZI and BRAF inhibitors induced cell death is NRF2 dependent. **(A,B)** Percentage viability of C32 **(A)** and SK-MEL-28 **(B)** cells upon treatment with diABZI and BRAFis, Dabrafenib (DF) and Vemurafenib (VF) using MTS cell viability assay (*n* = 3). **(C)** Percentage Cell viability upon treatment with diABZI and BRAFis in the presence of CDDO-Me (*n* = 3). ***P < 0.001.

Since we observed dying cells in the cell proliferation and migration assays upon diABZI treatment, we next asked if the combination of diABZI with BRAFi also induces cell death in melanoma cells. MTS viability assay performed on C32 and SK-MEL-28 cells treated with diABZI, diABZI + Dabrafenib, and diABZI + Vemurafenib revealed that diABZI induces cell-death which was significantly enhanced when combined with the BRAFis (Figures 5A,B). Interestingly, combination of diABZI with Vemurafenib was more potent in inducing cell-death in both the cell lines tested (Figures 5A,B). NRF2 activation protects cancer cells and promotes survival and repression of NRF2 was found to reverse drug resistance and sensitivity against the chemotherapeutic agents (23–25). Thus, we also questioned if the synergistic cell-death caused by diABZI in combination with BRAFis was dependent on NRF2. To study the effect of NRF2 in preventing cell-death we treated cells with CDDO-Me which is an NRF2 activator (22). Treatment with CDDO-Me significantly protected melanoma cells from diABZI induced cell death (Figure 5C). Taken together our data suggests that the STING agonist diABZI is highly potent in overcoming BRAFi generated drug resistance in melanoma cells by abating NRF2 activation.

## Discussion

**Figure 6:**
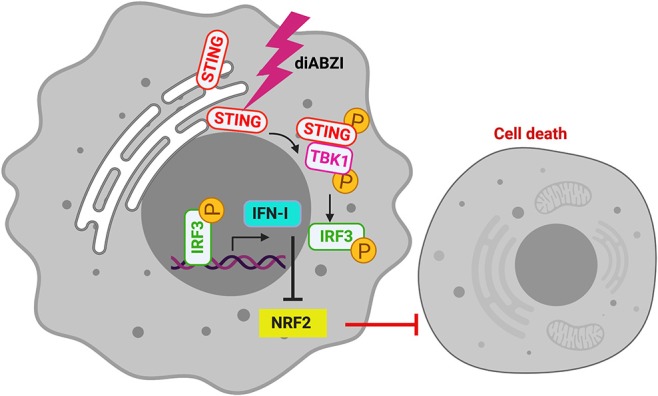
diABZI induced STING-activation and its downstream signaling abrogates NRF2 dependent cytoprotective responses resulting in cell death.

The role of STING in innate immune sensing and inflammation has been wel-documented (26, 27). This property of immune activation by STING is also beneficial in cancer therapy. Evidences suggest that STING activation in the tumor microenvironment primes immune cells to target cancer cells (28) leading to the development of STING agonists by several pharma and biotech firms (29). For the first time a non-CDN small molecule (diABZI) that binds and activates STING has been synthesized which has also been reported to have anti-tumor activity (18). Consistently, we show that activation of STING in melanoma cells using diABZI reduces proliferation (Figure 6). Furthermore, diABZI accelerates cell-death and prevents migration of melanoma cells when used in combination with BRAFis. Our data also reveals that diABZI synergizes with BRAFis in augmenting cell-death in melanoma cells by restricting NRF2.

In antigen-presenting cells (APCs) such as macrophages, STING upon binding to CDNs oligomerizes with TBK1 resulting in the phosphorylation of TBK1 and STING. This culminates in the activation of IRF3 and NF-kB to promote an adaptive T-cell response against cancer cells (30). However, STING signaling is muted in cancer cells including melanoma cells due to possible mutations in cGAS which converts double stranded DNA into CDNs (17). Pharmacological activation of STING with diABZI is independent of cGAS. Concurrently, we show that STING is phosphorylated which subsequently leads to the phosphorylation of TBK1 and IRF3 in melanoma cells when treated with diABZI. Intriguingly, total STING also decreased upon activation which is possibly due to degradation of STING through autophagy as diABZI treatment resulted in increased LC3B as shown previously (20).

We had recently shown that IFN-I signaling results in the abrogation of NRF2-dependent anti-oxidative responses leading to the death of macrophages during infection (10). As cGAS-STING is the primary pathway leading to IFN-I production, we asked if STING activation would prevent NRF2-response. Our observations suggest that diABZI-mediated STING activation decreases NRF2 expression and nuclear-translocation. The BRAF inhibitors which are currently used in treating melanoma, rewire metabolism and enhance ROS production. The altered metabolism helps melanoma cells to resist damage induced by ROS. NRF2 dependent anti-oxidative response is a vital mechanism employed by melanoma cells to survive oxidative stress (9). We now show that diABZI negatively regulates NRF2 in C32 and SK-MEL-28 melanoma cells and sensitizes them to death induced by BRAF inhibitors. Consistently, activation of NRF2 using CDDO-Me (22) protects melanoma cells from BRAFi/diABZI-induced cell death. In this context it is interesting to note that NRF2 has also been lately shown to negatively regulate STING during infection (31). Hence, increased NRF2 activation as we have observed could explain the defective STING activation reported in melanoma and other cancer cells.

Our data convincingly show that a combination of diABZI with BRAFis potently inhibit the proliferation, migration and viability of melanoma cells by disrupting NRF2. Although, Dabrafenib was developed as a more potent BRAFi than Vemurafenib (5), diABZI in synergy with Vemurafenib is more potent compared to its combination with Dabrafenib. The reduced ability of Dabrafenib in inducing cell death could be due to its ability to inhibit RIP3K (32) which, sequesters NRF2 in the cytosol by phosphorylating PGAM5 (10). Hence, we propose that combination of STING agonist such as diABZI and Vemurafenib as an effective therapeutic strategy to circumvent drug resistance in treating melanoma.

## Data Availability Statement

The datasets generated for this study are available on request to the corresponding author.

## Author Contributions

NR and SC: conceptualization, writing—original draft, and funding acquisition. NR, SC, and RG: methodology. SC and RG: investigation. NR, SC, SD, and MK: writing—review and editing. NR: resources and supervision.

## Conflict of Interest

The authors declare that the research was conducted in the absence of any commercial or financial relationships that could be construed as a potential conflict of interest.
