# Supplementary material for: Pharmacological STING Activation Is a Potential Alternative to Overcome Drug-Resistance in Melanoma
Source: Front Oncol. 2020 May 14;10:758. doi: 10.3389/fonc.2020.00758 (PMC7241280; doi:10.3389/fonc.2020.00758)
Supplement: Supplementary file 7 [file Data_Sheet_1.pdf]

Fig S1
(a) Representative immunoblot of LC3B upon diABZI treatment in C32 melanoma cells.

Fig S2
(a) Representative immunoblot of NRF2 upon diABZI and BRAFi treatment in SK-MEL-28 cells. (b) Immunofluorescence staining and microscopic examination of nuclear translocation of NRF2. Scale bar = 10µm.

Fig S3
(b) Quantification of time-lapse images acquired at 0, 1, 2, 6, 12 & 24 h of treatment versus untreated using ImageJ software.

Supplementary Video 1
Live imaging of untreated C32 cells

Supplementary Video 2
Live imaging of dabrafenib treated C32 cells

Supplementary Video 3
Live imaging of vemurafenib treated C32 cells

Supplementary Video 4
Live imaging of diABZI treated C32 cells

Supplementary Video 5
Live imaging of dabrafenib and diABZI treated C32 cells

Supplementary Video 6
Live imaging of vemurafenib and diABZI treated C32 cells
