# Supplementary figures and images for: Pharmacological STING Activation Is a Potential Alternative to Overcome Drug-Resistance in Melanoma
Source: Front Oncol. 2020 May 14;10:758. doi: 10.3389/fonc.2020.00758 (PMC7241280; doi:10.3389/fonc.2020.00758)

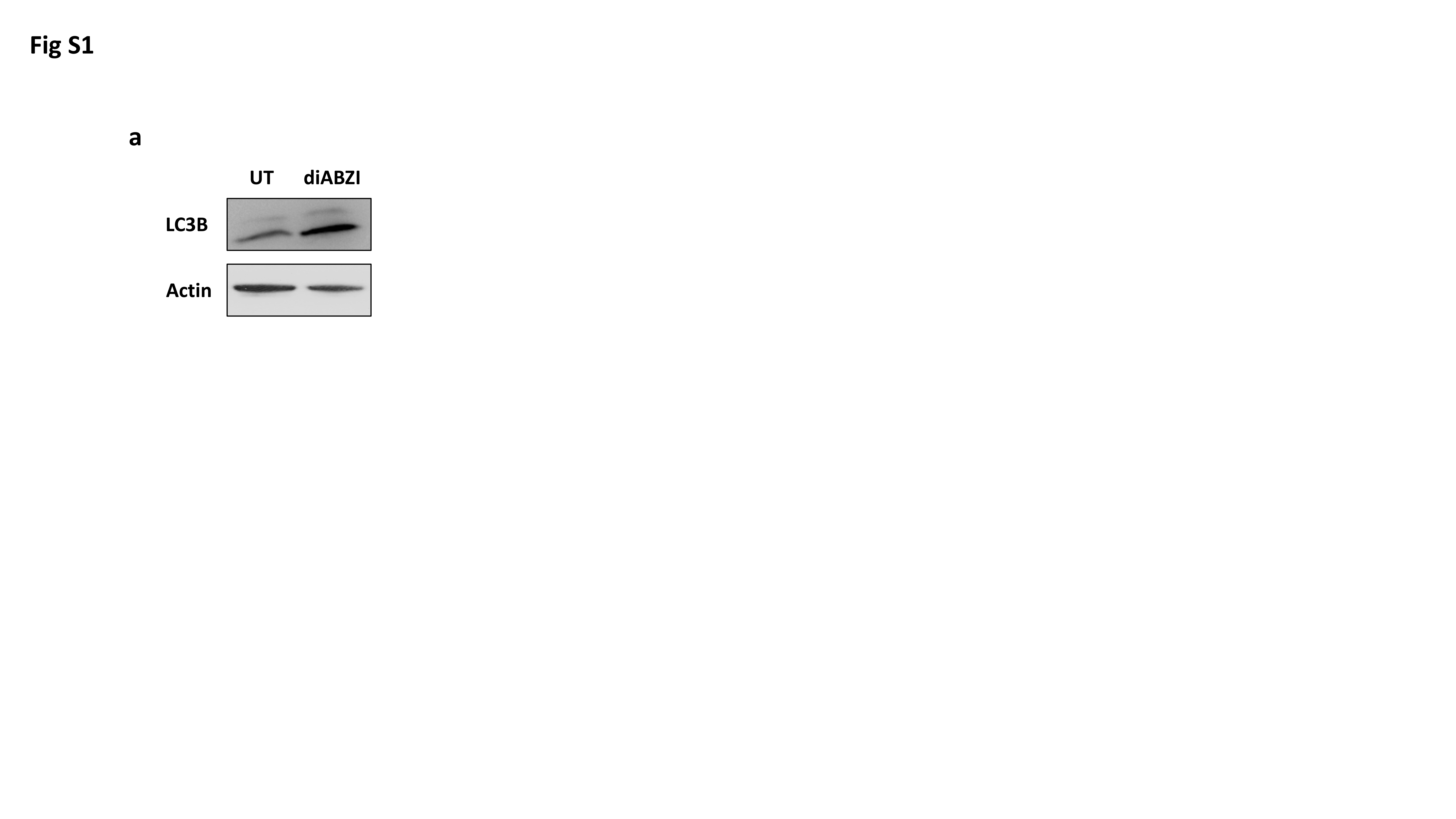

Supplement: Supplementary file 8 [file Image_1.tiff]

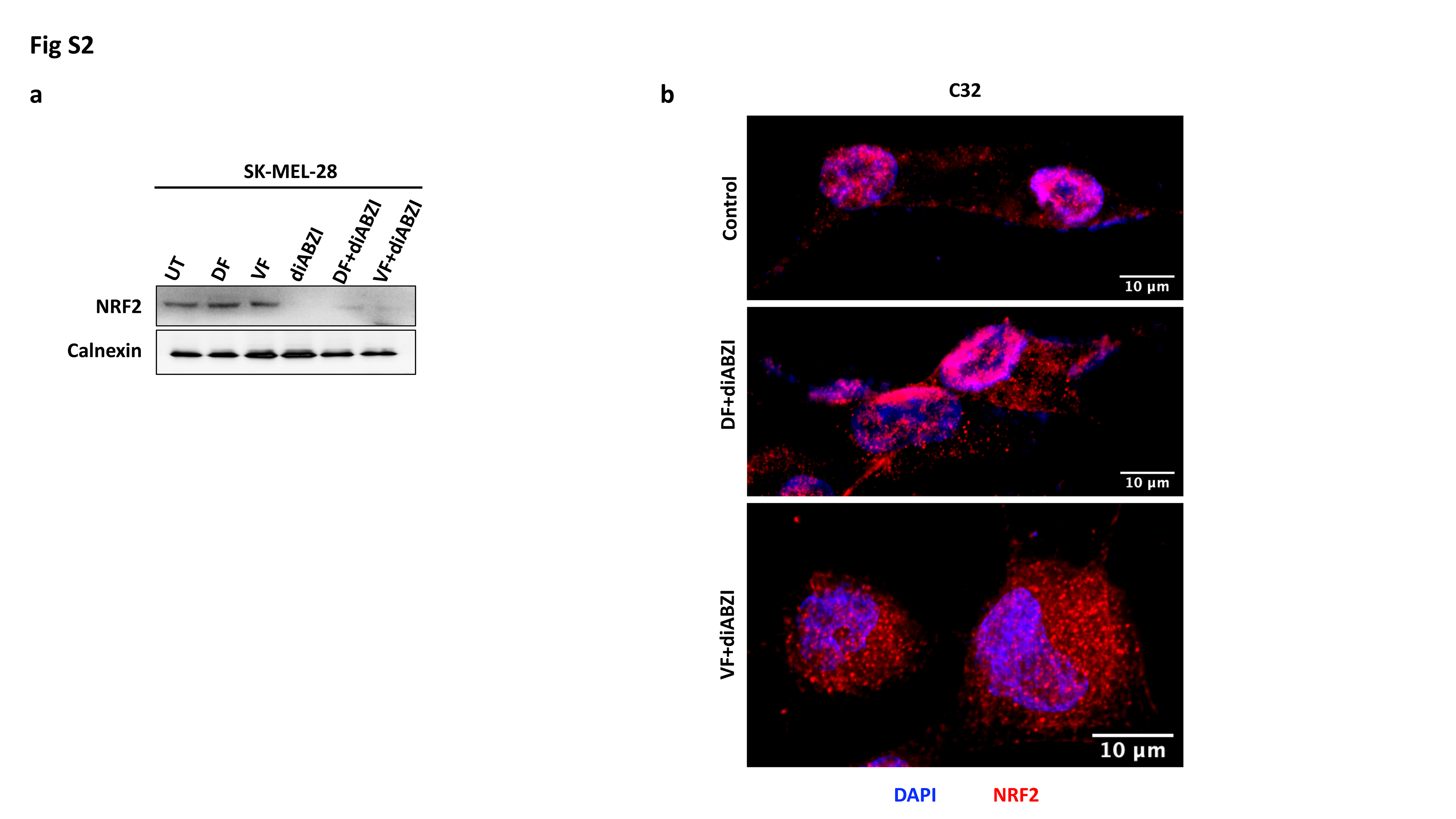

Supplement: Supplementary file 9 [file Image_2.tiff]

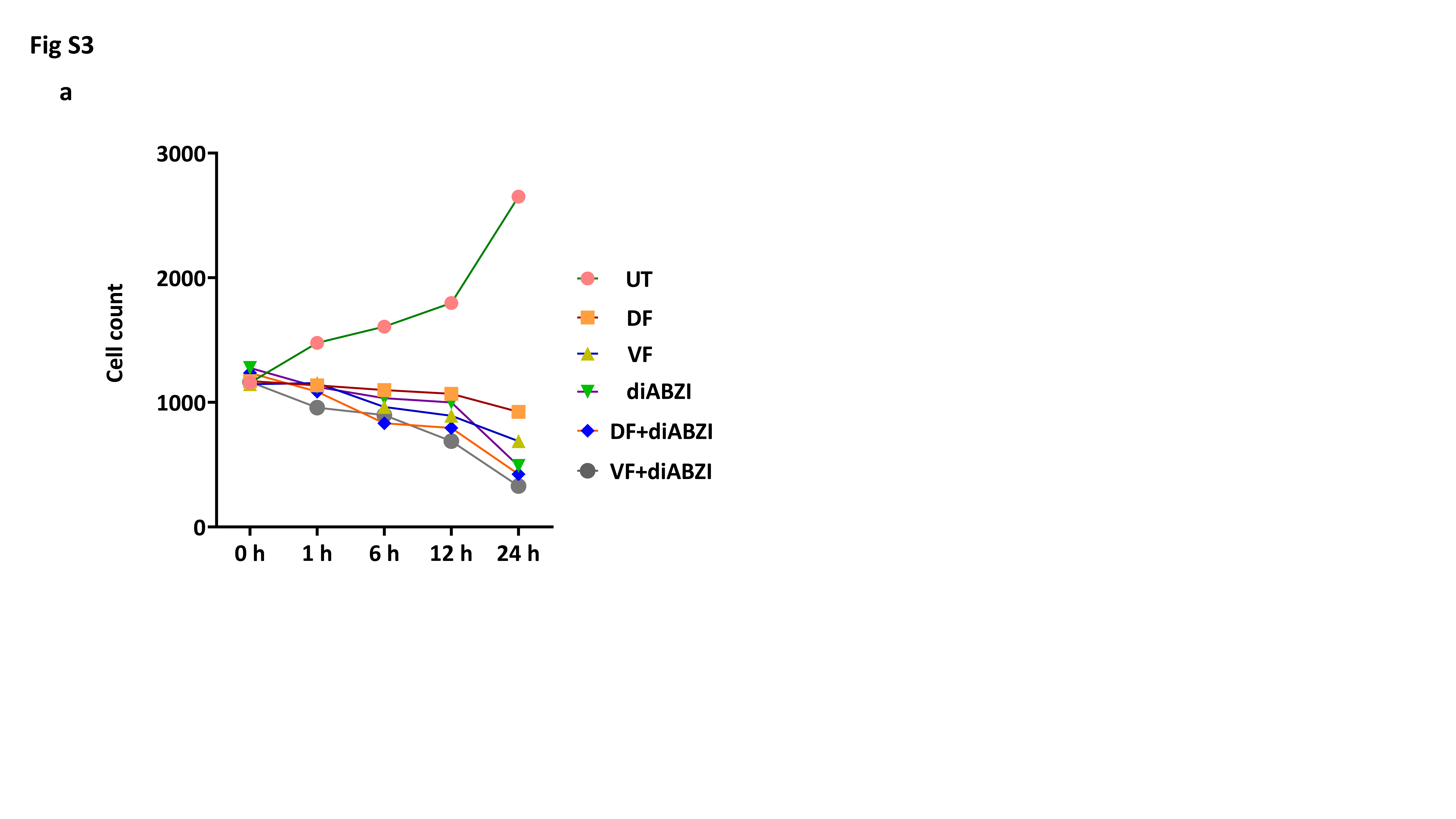

Supplement: Supplementary file 10 [file Image_3.tiff]
